# Effects of furosemide and tracer selection on urinary activity and peri-bladder artefacts in PSMA PET/CT: a single-centre retrospective study

**DOI:** 10.1186/s13550-022-00913-y

**Published:** 2022-07-27

**Authors:** Maarten L. Donswijk, Maurits Wondergem, Linda de Wit - van der Veen, Natascha M. Bruin, Pim J. van Leeuwen, Henk G. van der Poel, Marcel P. M. Stokkel, Wouter V. Vogel

**Affiliations:** 1 Department of Nuclear Medicine, Antoni van Leeuwenhoek Nederlands Kanker Instituut, Plesmanlaan 121, 1066 CX Amsterdam, the Netherlands; 2 Department of Radiation Oncology, Antoni van Leeuwenhoek Nederlands Kanker Instituut, Amsterdam, The Netherlands; 3 Department of Urology, Antoni van Leeuwenhoek Nederlands Kanker Instituut, Prostate Cancer Network Amsterdam, Amsterdam, The Netherlands; 4 Department of Urology, Amsterdam University Medical Center, Prostate Cancer Network Amsterdam, VU University, Amsterdam, The Netherlands

**Keywords:** PSMA, Furosemide, Sensitivity, Prostate cancer

## Abstract

**Background:**

High urinary activity in urinary bladder and ureters may hamper interpretation of prostate cancer and regional nodal metastases in prostate-specific membrane antigen (PSMA) PET/CT. The goal of this study was to assess effects of furosemide and choice of tracer on urinary activity in the bladder and ureters, as well as on occurrence of peri-bladder artefacts in PET/CT.

**Methods:**

Four cohorts with a total of 202 men staged with PSMA PET/CT for prostate cancer received either ^68^Ga-PSMA-11 as tracer, with (cohort G+) or without 10mg intravenous furosemide (G−) concurrent with tracer, or ^18^F-DCFPyL with (F+) or without furosemide (F−). SUVmax of bladder and ureters, presence, type, and severity of peri-bladder artefacts were compared between cohorts. The influence of furosemide and choice of tracer was determined while taking differences in biodistribution time into account.

**Results:**

Median SUVmax bladder was 43,5; 14,8; 61,7 and 22,8 in cohorts G−, G+, F− and F+, respectively, resulting in significant overall (*p *< 0.001) and between cohort differences (*p* adjusted < 0.001 to 0.003) except between G− and F+. Median SUVmax ureter was 6.4; 4.5; 8.1 and 6.0 in cohorts G−, G+, F− and F+, respectively, resulting in significant overall (*p *< 0.001) and between cohort differences for G+ : F− and F− : F+ (*p *< 0.001, respectively, 0.019). Significant effects of furosemide and choice of tracer on SUVmax bladder (*p *< 0.001 resp. *p *= 0.001) and of furosemide on SUVmax ureter (*p *< 0.001) were found, whereas differences in biodistribution time had not impacted these results significantly. Peri-bladder artefacts were present in 42/202 (21%) patients and were significantly more frequent in the F− cohort, respectively, less frequent in the G+ cohort (*p *= 0.001 resp. *p *< 0.001). Peri-bladder artefacts had a direct positive correlation with SUVmax bladder (*p *= 0.033).

**Conclusions:**

Increased urinary activity and higher incidence of peri-bladder artefacts were found in ^18^F-DCFPyL compared to ^68^Ga-PSMA-11 PET/CT. Effective reduction of urinary activity may be reached through forced diuresis using 10mg intravenous furosemide, which is especially advantageous in ^18^F-DCFPyL PET/CT.

## Background

Positron emission tomography/computed tomography (PET/CT) imaging targeting the prostate-specific membrane antigen (PSMA) plays an increasingly important role in the detection and staging of primary and recurrent prostate cancer (PCa) and has proven superior over conventional imaging methods [1, 2]. PSMA PET/CT for staging of prostate cancer requires optimal image quality, especially in the pelvis as primary area of interest for staging intermediate or high-risk primary prostate cancer and biochemical recurrent prostate cancer. Commonly used PSMA-ligands such as ^68^Ga-PSMA-11 and ^18^F-DCFPyL are excreted via the urinary tract into the bladder. High tracer concentration leading to intense signal activity in the bladder can compromise image quality and interpretation of PET scans in the adjacent prostate bed by visual obscurement of lesions or introduction of artefacts (e.g. halo-artefacts) surrounding the bladder [3, 4]. Similarly, increased activity in the ureters may compromise image quality and assessment of pelvic lymph node status. Commonly applied strategies to improve clearance of accumulated tracer from the ureters and bladder and to increase tumour-to-background visibility are oral or intravenous hydration and forced diuresis [4–9] which is advocated in the joint EANM and SNMMI guidelines for ^68^Ga-PSMA-11 [10].

In our centre from 2018 onward, ^68^Ga-PSMA-11 and ^18^F-DCFPyL, which have similar biokinetic profiles [11], have been used concurrently in clinical PSMA PET/CT imaging for reasons of availability and logistics. Both tracers were applied in conjunction with oral hydration and forced diuresis using intravenous furosemide. However, in a population of men that often experience some degree of urine incontinence (post-radical prostatectomy), the combination of extra hydration and forced diuresis can be problematic and may lead to patient discomfort. Due to frequent problems with urinary urge complaints and urine contamination, it was decided in our department to stop the administration of furosemide before PSMA PET/CT. However, this raised the question which effect this discission had on urinary activity and peri-bladder artefacts in PSMA PET/CT and if this would be of the same magnitude for different PSMA tracers. In this study, we present the results of a retrospective evaluation on the effects of furosemide (e.g. forced diuresis) and the type of tracer (^68^Ga-PSMA-11 versus ^18^F-DCFPyL) on the urinary activity in the bladder and ureters, as well as on the occurrence, type and severity of peri-bladder artefacts.

## Methods

### Patient cohorts

This single-centre retrospective data analysis study was approved by the Institutional Review Board (IRBd20-103) of the Netherlands Cancer Institute (Amsterdam, The Netherlands). Imaging data were selected of consecutive patients who underwent a PSMA-ligand PET/CT using ^68^Ga-PSMA-11 or ^18^F-DCFPyL as tracer between June 2016 and March 2020. Indications for PSMA PET/CT were either staging of intermediate/high-risk primary PCa (≥ cT3, ≥ 4+3 (or ISUP > 2) or PSA ≥ 20 ng/mL) or restaging of biochemical recurrent PCa after treatment with curative intent. First, protocols were employed with forced diuresis using furosemide administration for all patients. Later on, protocols changed and no administration of furosemide was employed in any of the patients. In order to include around two hundred evaluable patients, four cohorts were created by selecting the first sixty consecutive patients from each unique protocol combination. These cohorts were defined as: ^68^Ga-PSMA-11, without furosemide (G− ); ^68^Ga-PSMA-11, with furosemide (G+); ^18^F-DCFPyL, without furosemide (F−); ^18^F-DCFPyL, with furosemide (F+).

Patients with prior cystectomy, a urinary bladder catheter, incomplete or externally acquired imaging data, incorrect tracer registration, or incomplete data regarding furosemide administration were excluded from the study.

### Radiopharmaceutical and imaging protocol

PSMA PET/CT imaging was performed using a Philips Gemini TF‐II (Philips Healthcare®, the Netherlands/USA). ^68^Ga-Glu-urea-Lys(Ahx)-HBED-CC ([^68^Ga]Ga-PSMA-11) was radiolabeled in-house using a fully automated system (Scintomics GmbH, Germany). Scanning commenced approximately 45 min post injection (PI). In cohort G+ administered tracer dose was approximately 100MBq combined with acquisition times of 3 min per bed position (min/bp) for the pelvis and 2 min/bp for the remainder of the scan range. After a national consensus meeting, the clinical protocol for ^68^Ga-PSMA PET/CT imaging in our centre changed. Therefore, in cohort G− administered tracer dose was approximately 150MBq combined with acquisition times of 4.0 min per bed position (min/bp) for the pelvis and 2.5 min/bp for the remainder of the scan range.

2-(3-{1-carboxy-5-[(6-[^18^F]fluoro-pyridine-3-carbonyl)-amino]-pentyl}-ureido)-pentanedioic acid (^18^F-DCFPyL) purchased through BV Cyclotron (Amsterdam, the Netherlands) was administered as an intravenous bolus injection with a fixed dose of 200 MBq. Scanning commenced approximately 60 min PI, with 2 min/bp over the complete scan range (cohorts F− and F+).

**Fig. 1:**
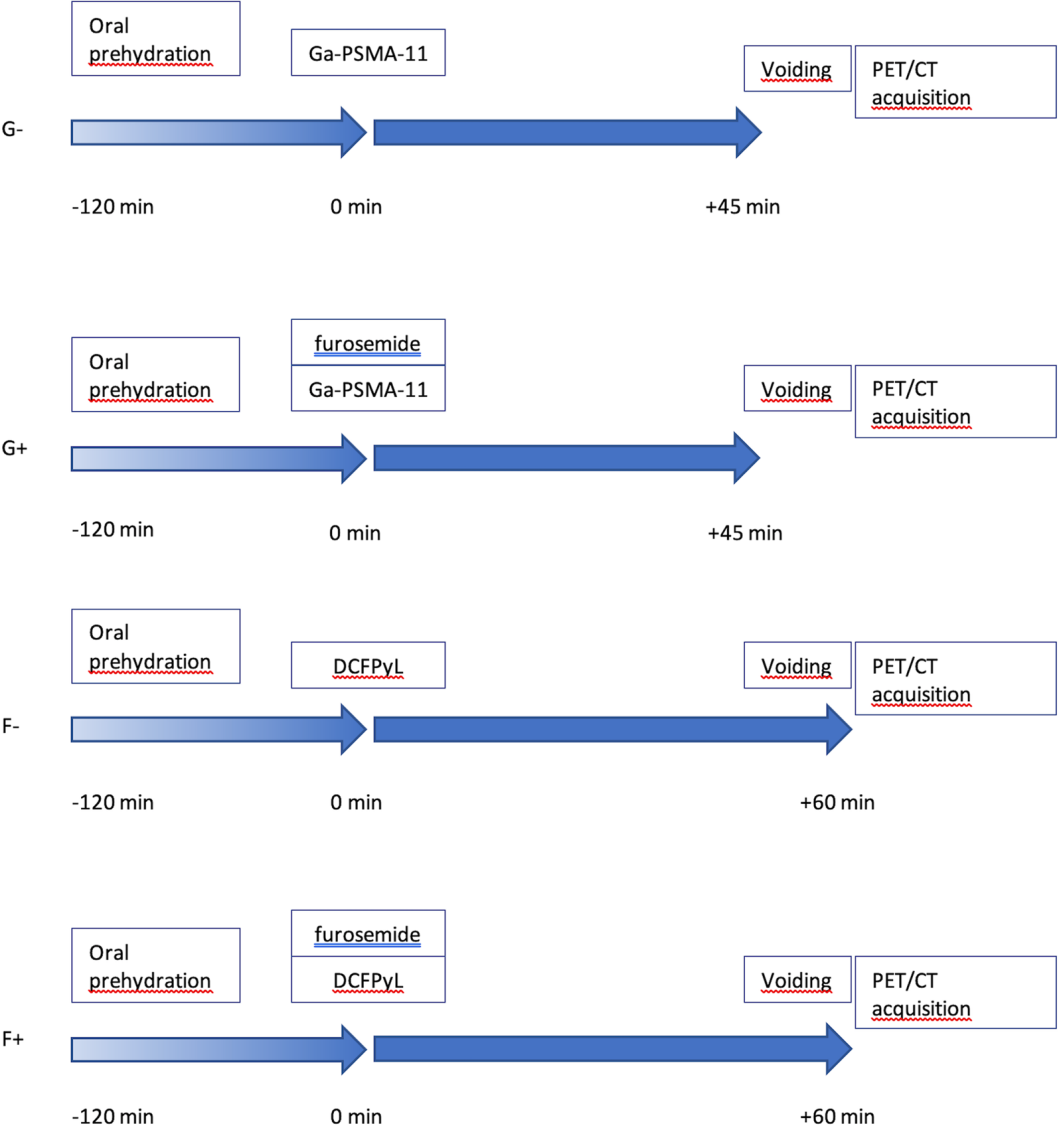
Patient preparation and scanning protocol per cohort. ^68^Ga-PSMA-11, no furosemide (G−); ^68^Ga-PSMA-11 and furosemide (G+); ^18^F-DCFPyL, no furosemide (F−); ^18^F-DCFPyL and furosemide (F+).

In cohorts G+ and F+, 10mg intravenous furosemide was administered per protocol to patients as an intravenous bolus concurrently with the tracer. Independent of tracer, patients were instructed to drink at least 0.5L of water within two hours before tracer injection, and to void just before start of scanning (Fig. 1). Data were reconstructed using an iterative OSEM-3D algorithm: three iterations, 33 subsets, no filter. Image matrix size was 144 × 144, pixel spacing 4 × 4 mm and slice thickness 4 mm. PET-images were combined with a low-dose CT (120–140 kV, 40–80 mAs with dose modulation) for attenuation correction and anatomical reference. All PET images were corrected for scatter, decay, and random coincidences.

### Data acquisition

**Fig. 2:**
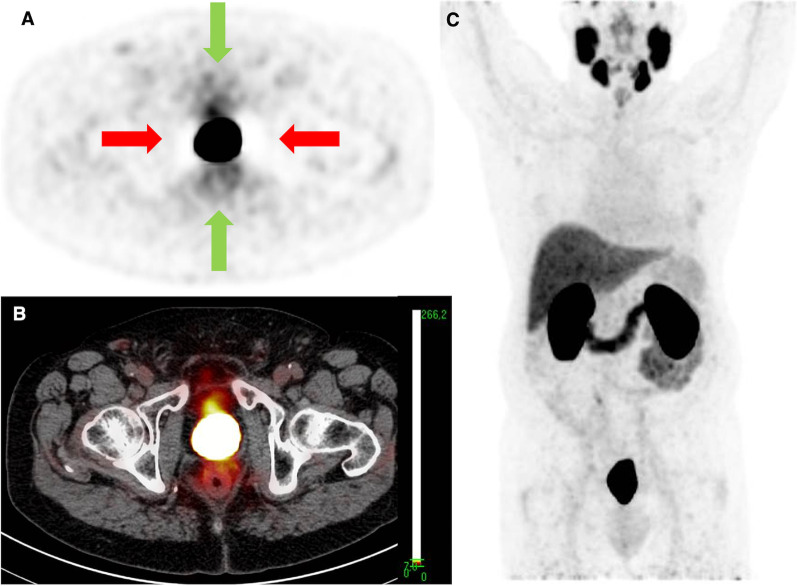
PSMA PET/CT with ^18^F-DCFPyL used as tracer in a 68 years old man staged for staging of biochemical recurrence of prostate cancer showing peri-bladder artefacts, deteriorating image quality and impairing image interpretation. **A** Axial PET reconstructions; **B** fused axial PET/CT reconstructions; and **C** PET maximum intensity projection (MIP). Window width SUV range 0–7 applied. Red arrows: PET photopenic area (halo artefact). Green arrows: increased PET activity (flare artefact).

All images were analysed using the Osirix® Dicom Viewer (Pixmeo, Geneva, Switzerland). By manually placing a volume of interest (VOI) in the bladder over the hottest region, the maximum standardized uptake value (SUVmax bladder) corrected for bodyweight of the urinary activity of ^68^Ga-PSMA-11 and ^18^F-DCFPyL was determined. The presence or absence of visually discernable activity (SUVmax ≥ 3.0, i.e. around two times SUVmax of bloodpool activity) in one or more ureters was noted. If present, again SUVmax was measured by placing a VOI over the hottest region (SUVmax ureter). Furthermore, the presence and type of PET artefacts surrounding the bladder were noted: (1) halo-artefacts, defined as areas with artefactual photopenia surrounding the bladder and/or (2) flare-artefacts, defined as areas with artefactual increased activity surrounding the bladder, as well as the severity of the artefact: (1) mild (visible but not interfering with visual interpretation) or (2) severe: strongly present, interfering with visual interpretation (Fig. 2).

To investigate the possible influence on PET image noise characteristics of differences in tracer dosages and PET acquisition times among the cohorts, ten random patients were chosen from every cohort (forty in total) and a 3.0 cm VOI was placed within the right liver lobe, which generally is an area of diffuse, homogenous physiological tracer uptake. Within this VOI, the standard deviation of the standardized uptake value (SUV_SD) was determined, which may serve as an indicator of image noise.

### Statistical analysis

Continuous variables are expressed as mean (± SD), or in case of not normally distributed data as median (IQR). Categorical variables are presented with absolute and relative frequencies. Baseline clinical differences between the cohorts were tested using a one-way analysis of variance (ANOVA).

The Kruskal–Wallis (KW) test was used to test for the presence of an overall difference between the cohorts in SUVmax bladder, SUVmax ureter and SUV_SD, whereafter Dunn’s post hoc pairwise comparisons with Bonferroni adjustment were carried out to determine which cohorts deviated. To analyse the effects of furosemide and the choice of tracer on SUVmax bladder and SUVmax ureter, linear regression analyses were performed using SUVmax as dependent variable and furosemide and choice of tracer as independent variables, together with biodistribution time to take into account the differences in biodistribution time between tracers.

Fisher’s exact test for independence was used to test for overall differences between cohorts in the presence of visual ureteral activity and peri-bladder artefacts, followed by post hoc pairwise Z-tests with Bonferroni adjustment. To analyse the effects of furosemide and choice of tracer on the presence or absence of ureteral activity and peri-bladder artefacts, a logistic regression analysis was performed. Significance level was set at α *.*05. All analyses were carried out using Statistical Package for Social Sciences (SPSS, IBM; v27).

## Results

From the 240 selected patients, 38 were excluded because of incorrect tracer registration (*n* = 18), incomplete data regarding furosemide administration (*n* = 12), incomplete (*n* = 3) or external imaging data (*n* = 3), prior cystectomy (*n* = 1), or a urinary bladder catheter (*n* = 1). In total, 202 patients were included. Relevant baseline characteristics are displayed per cohort in Table 1. Between cohorts there were no significant differences in patients characteristics (age, weight and kidney function).

**Table 1:** Patient characteristics per cohort (total *n *= 202)

	Cohorts				*P* value
	G− (*n *= 51)	G+ (*n*=50)	F− (*n *= 51)	F+ (*n *= 50)	
Age (years), mean ± SD	69.1 ± 7.0	70.3 ± 6.9	69.2 ± 7.2	69.2 ± 6.5	0.779
Weight (kg), mean ± SD	88.5 ± 12.5	85.7 ± 12.9	88.3 ± 14.5	84.3 ± 10.9	0.503
GFR (ml/min/1.73 m^2^), mean ± SD	79.6 ± 16.9	77.9 ± 16.9	74.5 ± 18.2	74.9 ± 17.8	0.476
Administered activity (MBq), mean ± SD	148.6 ± 11.1	90.9 ± 8.9	194.5 ± 12.9	233.4 ± 65.8	**< 0.001**
Biodistribution time (min), mean ± SD	48.7 ± 5.3	49.0 ± 7.2	64.5 ± 6.2	59.4 ± 8.8	**< 0.001**

Bold values are significant

GFR, glomerular filtration rate; SD, standard deviation

*p values for overall cohort comparison from one-way ANOVA

### Urinary bladder activity

Median (IQR) SUVmax bladder was 43.5 (49.7), 14.8 (13.9), 61.7 (83.1), and 22.8 (20.5) in cohorts G−, G+, F−, and F+, respectively, resulting in a significant difference for overall group comparison (KW test; *p *< 0.001) (Table 2). Pairwise comparisons (Dunn’s test) resulted in significant differences between all cohorts (*p* adjusted < 0.001 to 0.003*)* except between G− and F+.

**Table 2:**
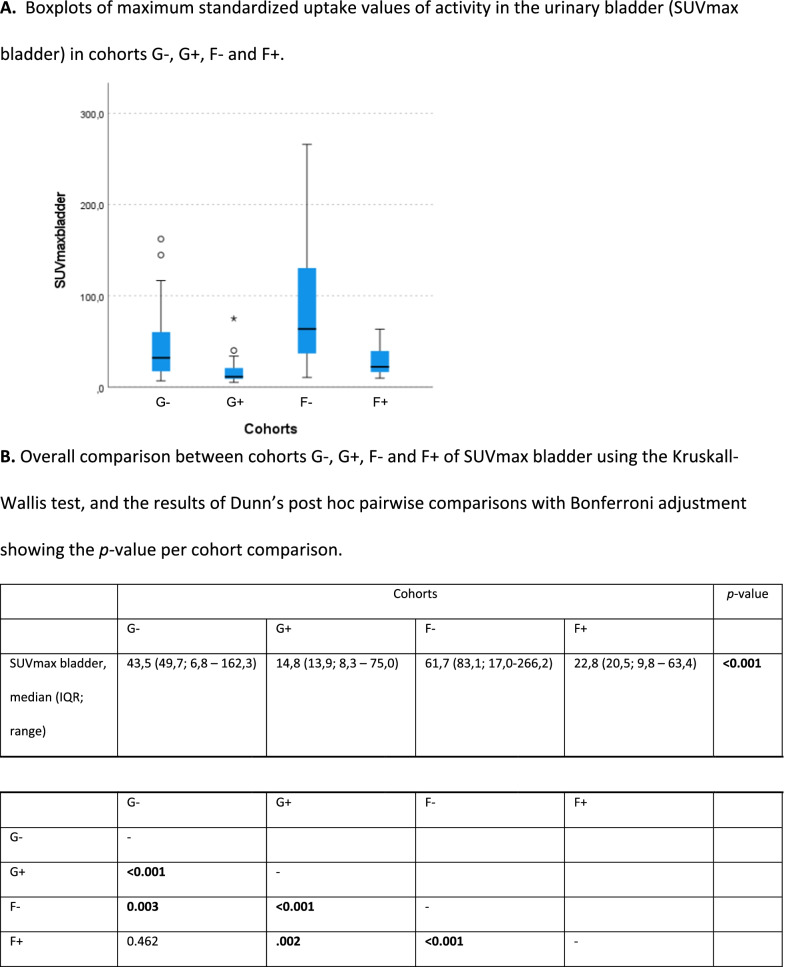
A. Boxplots of maximum standardized uptake values of activity in the urinary bladder (SUVmax bladder) in cohorts G-, G+, F- and F+. B. Overall comparison between cohorts G-, G+, F- and F+ of SUVmax bladder using the Kruskall-Wallis test, and the results of Dunn’s post hoc pairwise comparisons with Bonferroni adjustment showing the p-value per cohort comparison

Bold values are significant

IQR = interquartile range

To correct for non-normality, SUVmax bladder values were log transformed and were used as dependent variable in the regression analyses. A significant effect of furosemide (*p *< 0.001) and choice of tracer (*p *= 0.001) on SUVmax bladder was found, whereas no significant effect of biodistribution time on SUVmax bladder was found (Table 3A).

**Table 3:** Linear regression analyses assessing the effects of administration of furosemide, choice of tracer and biodistribution time on SUVmax bladder (A) and SUVmax ureter (B)

	B	SE	Exp(B)	*P* value
*A. SUVmax bladder*				
Variables				
Radiopharmaceutical	0.213	0.065	1.237	**0.001**
Furosemide	− 0.414	0.044	0.661	**< 0.001**
Biodistribution time	0.005	0.004	1.005	0.159
*B. SUVmax ureter*				
Variables				
Radiopharmaceutical	0.075	0.066	1.078	0.257
Furosemide	− 0.188	0.043	0.829	**< 0.001**
Biodistribution time	0.003	0.004	1.003	0.409

Bold values are significant

B, b-coefficient; SE, standard error for b-coefficient; and Exp(B), exponentiated b-coefficient

### Quantitative ureteral activity

Median (IQR) SUVmax ureter was 6.4 (7.6), 4.5 (2.5), 8.1 (12.9), and 6.0 (5.1) in cohorts G−, G+, F−, and F+, respectively, resulting in a significant difference for overall group comparison (KW test; *p * < 0.001) (Table 4). Pairwise comparisons (Dunn’s test) resulted in significant differences only between cohorts G+ and F− (*p* adjusted < 0.001) and between F− and F+ (*p* adjusted 0.019).

**Table 4:**
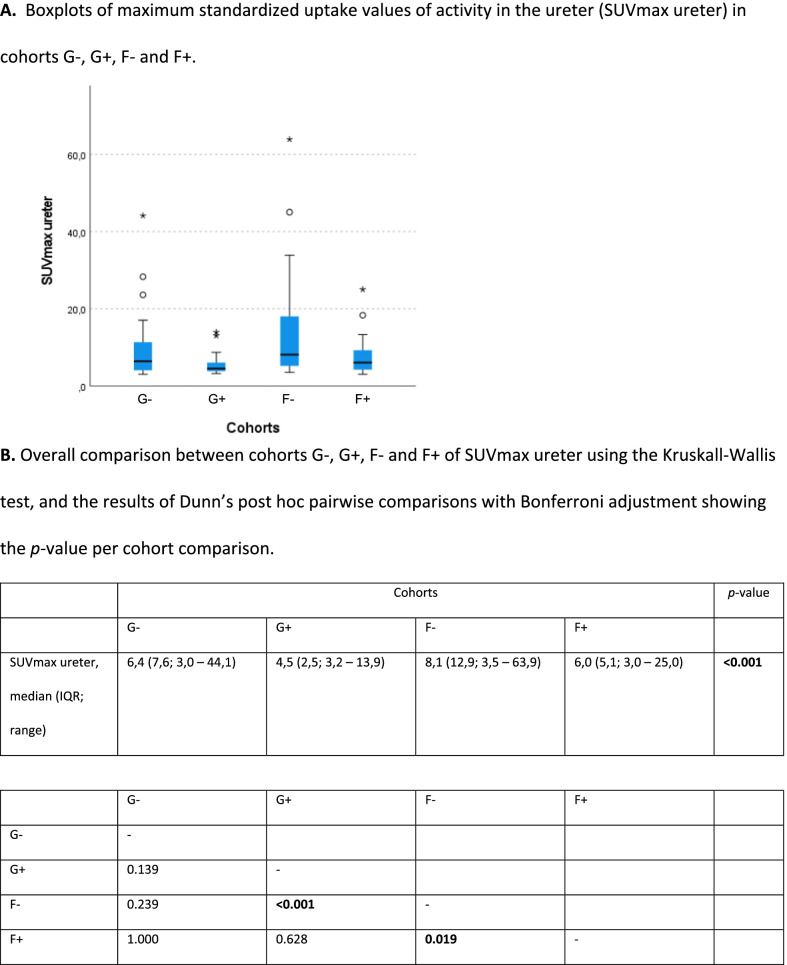
A. Boxplots of maximum standardized uptake values of activity in the ureter (SUVmax ureter) in cohorts G-, G+, F- and F+. B. Overall comparison between cohorts G-, G+, F- and F+ of SUVmax ureter using the Kruskall-Wallis test, and the results of Dunn’s post hoc pairwise comparisons with Bonferroni adjustment showing the p-value per cohort comparison

Bold values are significant

IQR = interquartile range

To correct for non-normality, SUVmax ureter values were log transformed and were used as dependent variable in the regression analyses. A significant effect of furosemide on SUVmax ureter was found (*p * < 0.001), whereas no significant effect of choice of tracer and biodistribution time on SUVmax ureter was found (Table 3B).

### Visual ureteral activity

Fisher’s exact test indicated a significant difference in the incidence of visually discernable activity in one or both of the ureters across the cohorts (χ^2^(3, *N  *= 202) = 9.42, *p * = 0.024). However, on the cohort-level pairwise Z-tests with correction for multiple testing did not show significant differences (*p * = 0.116–0.674) (Table 5A).

**Table 5:** A. Observed frequencies (n) and overall differences (Fisher’s exact test) in incidence of visually discernable activity in in the ureters between the cohorts. Results from post hoc pairwise Z-tests corrected for multiple testing are shown as *p*-values per cohort. B. Logistic regression analysis assessing the relation between administration of furosemide or choice of tracer on the incidence of visually discernable activity in the ureters

A. Observed frequencies (n) and overall differences (Fisher’s exact test) in incidence of visually discernable activity in in the ureters between the cohorts. Results from post hoc pairwise Z-tests corrected for multiple testing are shown as *p*-values per cohort						
		G−	G+	F−	F+	Totals
Visual ureter activity	Yes	36	33	44	43	156
	No	15	17	7	7	46
*P* value		0.764	0.116	0.299	0.353	

B. Logistic regression analysis assessing the relation between administration of furosemide or choice of tracer on the incidence of visually discernable activity in the ureters				
	B	SE	Odds ratio (95% CI)	*P* value
*Variables*				
Radiopharmaceutical	0.963	0.510	2.619 (0.964–7.115)	0.059
Furosemide	− 0.401	1.032	0.669 (0.088–5.064)	0.697
*Interaction effects*				
Radiopharmaceutical * furosemide	0.189	0.718	1.208 (0.296–4.934)	

B, b-coefficient; SE, standard error for b-coefficient; and CI, confidence interval

Overall differences between cohorts *p * = 0.025

On logistic regression analysis, no significant effect of administration of furosemide or choice of tracer on the incidence of visually discernable activity in one or both ureters was found (Table 5B).

### Peri-bladder artefacts

Peri-bladder artefacts were present in 42/202 (21%) patients. Fisher’s exact test indicated a significant difference in the incidence of peri-bladder artefacts across the cohorts (χ^2^(3, *N *= 202) = 41.60, *p *< 0.001). Pairwise Z-tests with correction for multiple testing showed a significantly lower incidence of peri-bladder artefacts in the G+ (*p < *0.001) and a higher incidence in the F− cohort (*p = *0.001), whereas the G− (*p = *1.000) and F+ cohorts (*p = *0.310) did not significantly differ from the other cohorts (Table 6A).

**Table 6:** A. Observed frequencies (*n*) and overall differences (Fisher’s exact test) in incidence of peri-bladder artefacts between the cohorts. Results from post hoc pairwise Z-tests with Bonferroni correction for multiple testing are shown as *p *values per cohort. B. Concordance of incidence, type, and severity of peri-bladder artefacts. C. Logistic regression analysis assessing the relation between administration of furosemide or choice of tracer on the incidence of peri-bladder artefacts

A. Observed frequencies (*n*) and overall differences (Fisher’s exact test) in incidence of peri-bladder artefacts between the cohorts. Results from post hoc pairwise Z-tests with Bonferroni correction for multiple testing are shown as *p *values per cohort							
		Type, severity	G−	G+	F−	F+	totals
Peri-bladder artefacts	Yes (any)		9	1	26	6	42
		Halo, mild	*6*	*1*	*8*	*2*	*17*
		Halo, severe	*1*	*0*	*14*	*0*	*15*
		Flare, mild	*8*	*1*	*10*	*6*	*25*
		Flare, severe	*1*	*0*	*14*	*0*	*15*
	No		42	49	25	44	160
*P* value			1.000	**0.001**	**< 0.001**	0.310	

B. Concordance of incidence, type, and severity of peri-bladder artefacts				
Peri-bladder artefacts		Halo		
		No	Mild	Severe
Flare	No	160	2	0
	Mild	10	13	2
	Severe	0	2	13

C. Logistic regression analysis assessing the relation between administration of furosemide or choice of tracer on the incidence of peri-bladder artefacts				
	B	SE	Odds ratio (95% CI)	*P* value
*Variables*				
Radiopharmaceutical	1.887	1.296	6.602 (0.520–83.760)	0.145
Furosemide	− 1.500	1.348	0.223 (0.016–3.130)	0.266
SUVmax bladder	0.074	0.035	1.077 (1.006–1.153)	**0.033**
*Interaction effects*				
SUVmax bladder * radiopharmaceutical	− 0.017	0.019	0.983 (0.947–1.021)	0.378
SUVmax bladder * furosemide	0.038	0.029	1.038 (0.981–1.099)	0.196

Bold values are significant

Italic numbers are subcategories of "Yes (any)"

Overall differences between cohorts *p *< 0.001

B, b-coefficient; SE, standard error for b-coefficient; CI, confidence interval

The 42 peri-bladder artefacts included 32 halo-artefacts (17 mild, 15 severe) and 40 flare-artefacts (25 mild, 15 severe). In 30 out of 42 peri-bladder artefact cases, both flare- and halo-artefacts occurred together. Furthermore, in these cases the severity of the artefacts was closely linked: 26 cases showed similar severity, four cases dissimilar severity, and all cases with a severe artefact of one type showed an artefact of the other type as well (Table 6B).

On logistic regression analysis, a significant effect of SUVmax bladder on incidence of peri-bladder artefacts was found (*p *= 0.033), whereas no significant effect of administration of furosemide or choice of tracer was found (Table 6C).

### Image noise-level analysis

Median (IQR) SUV_SD was 0.49 (0.19), 0.89 (0.50), 0.48 (0.16), and 0.44 (0.16) in cohorts G−, G+, F−, and F+, respectively, resulting in a significant difference for overall group comparison (KW test; *p *= 0.003). Pairwise comparisons (Dunn’s test) resulted in significant differences between cohorts G+ and G− (*p* adjusted 0.043) and between G+ and F+ (*p* adjusted 0.005).

## Discussion

PSMA PET/CT has proven to be a valuable tool in staging of primary and recurrent PCa [1, 2]. The commonly used PSMA-ligands ^68^Ga-PSMA-11 and ^18^F-DCFPyL are excreted mainly via the urine. Difficulties in the assessment of structures adjacent to the urinary bladder and ureters have been recognized early in the clinical use of these tracers [3–5, 12]. In a recent study by Uprimny et al., it was shown in 220 patients that patient preparation with hydration and forced diuresis using furosemide prior to ^68^Ga-PSMA-11 PET/CT significantly increased the detection rate of local recurrence of PCa [9]. This strategy to improve clearance of accumulated tracer from the ureters and bladder was already advocated by the joint EANM and SNMMI procedure guideline for PCa imaging using ^68^Ga-PSMA-ligands [10]. It is likely that imaging using ^18^F-based PSMA tracers with high urinary excretion, such as ^18^F-DCFPyL, would benefit from such strategies as well. However, to date there are neither reports assessing the effects of forced diuresis in ^18^F-DCFPyL PET/CT nor reports describing a direct comparison between ^68^Ga and ^18^F-based PSMA tracers on urinary activity and peri-bladder artefacts in a large patient cohort.

The present data show that administration of intravenous furosemide concurrent with tracer injection considerably reduces tracer concentration in the urinary bladder in patients staged with PSMA PET/CT for prostate cancer, which is true for both ^68^Ga-PSMA-11 as well as ^18^F-DCFPyL. For ^68^Ga-PSMA-11, similar findings have been described in earlier studies [4, 7–9]]. In addition, extreme SUVmax bladder values (e.g. SUVmax bladder > 100) were not noted in the furosemide cohorts, but not infrequent in the other cohorts. Significantly higher urinary bladder activities were found in ^18^F-DCFPyL compared to ^68^Ga-PSMA-11 for the respective subgroups with and without furosemide. Theoretically the difference in timepoint of acquisition between the ^18^F-DCFPyL cohorts and the ^68^Ga-PSMA-11 cohorts (60 and 45 min PI, respectively) could, by continuing urinary excretion of tracer in that timeframe, contribute to higher urinary activity in the ^18^F-DCFPyL cohorts [13]. However, the observed differences in biodistribution time did not lead to a significant effect in the multivariate analysis, leaving choice of tracer and furosemide as independent factors influencing urinary bladder activity. A stronger effect of furosemide on urinary bladder activity was found in ^18^F-DCFPyL compared to ^68^Ga-PSMA-11, although the relative reduction by furosemide for both tracers was of the same magnitude (+/- 70%). Comparable reductions in urinary bladder activity for ^68^Ga-PSMA-11 were found by Fennesy et al. and Uprimny et al. administering 20mg intravenous furosemide concurrent with the tracer [4, 7]. Uprimny et al. reported an even greater reduction compared to a protocol without any patient preparation (no hydration, no furosemide). Therefore, hydration, which is already a standard procedure in many centres, seems a sensible measure to decrease urinary bladder activity [4, 7]. If there is a net benefit of intravenous compared to oral hydration is not clear.

Given the comparable noise levels for the G- and F cohorts, reported differences in SUVmax between these cohorts may not be explained by differences in tracer dosages and acquisition time. The G+ cohort, however, displayed larger SUV_SD values which may have led to increased SUVmax values of urinary tracer activity as well. However, the G+ cohort already displayed the lowest SUVmax values compared to the other cohorts, and these may have even been lower; therefore, reported effects and correlations in this study may even have been underestimated.

The effects of furosemide and choice of tracer on the ureteral activity were comparable to the effects on urinary bladder activity, although less pronounced. Visual ureteral activity was significantly different on the overall cohort level, but a clear relation of the incidence of visual ureter activity with administration of furosemide or choice of tracer was not found. On the other hand, SUVmax ureter was significantly influenced by both furosemide and choice of tracer and was shown to be significantly higher in the ^18^F-DCFPyL cohort without furosemide. These results are in line with earlier studies reporting a significant reduction of ureteral activity after administration of furosemide using ^68^Ga-PSMA-11 [4, 7] or ^68^Ga-PSMA-I&T as tracer [5].

Peri-bladder artefacts with a photopenic ‘halo-like’ area surrounding the bladder or kidneys were first described in ^68^Ga-PSMA-11 PET/MRI and recognized as impairing image interpretation [14]. This phenomenon is present in PET/CT as well and has also been described using ^18^F-DCFPyL as tracer [6]. In an experimental setting, these artefacts were found to be related to high contrast to background ratios and not tracer- or modality-dependent (PET/CT vs. PET/MRI) [15]. In this study, we found peri-bladder artefacts in up to fifty per cent of patients in the ^18^F-DCFPyL without furosemide cohort, though remarkably lower percentages in the ^68^Ga-PSMA-11 cohorts which is in line with earlier studies [4, 7]. We found a great overlap in occurrence and severity of halo- and flare-type peri-bladder artefacts, and this may well be explained by a common cause: erroneous scatter correction [15, 16]. This study confirms in a clinical setting that peri-bladder artefacts are encountered in ^68^Ga-PSMA-11 as well as ^18^F-DCFPyL PET/CT and that they are solely dependent on urinary bladder activity. Although the occurrence and severity of peri-bladder artefacts may be diminished by improved PET scatter correction algorithms [15, 16], these usually are an integrated part of the reconstruction algorithms provided by the manufacturers and not easily adjusted [7]. Therefore, strategies to decrease urinary bladder activity are also sensible to decrease the occurrence of peri-bladder artefacts. Late pelvic imaging has been reported as a strategy to decrease urinary bladder activity as well; however, this strategy lacks a clear benefit, while it complicates logistics [5, 6, 8].

The administration of furosemide concurrent with the tracer has no effect on physiologic uptake in ^68^Ga-PSMA-11 PET/CT, and thus, a negative impact of early furosemide injection on targeting properties and biodistribution of ^68^Ga-PSMA-11 seems unlikely [9]. Given the similarities in biodistribution of ^68^Ga-PSMA-11 and ^18^F-DCFPyL, these findings probably apply to ^18^F-DCFPyL as well. In our study, the effects on urinary activity and peri-bladder artefacts were observed using a relatively low dose of 10mg intravenous furosemide, whereas earlier studies used 20–40 mg furosemide as standard dose [4–9]. The frequency and severity of urinary urge complaints are likely related to the furosemide dose, whereas a higher dose may not increase the aimed effects of forced diuresis (7). Therefore, the optimal furosemide dose should be a balance of maximizing the aimed effects of forced diuresis on the one hand and minimizing the urinary urgency complaints on the other hand.

A limitation of this study is its retrospective nature. Different protocols have been used sequentially, and patient population may have varied over time. However, we assume these factors may have had only limited influence on the measured urinary activity and occurrence of peri-bladder artefacts. Furthermore, the clinical outcome of patients has not been evaluated in this study.

This study shows that in patients undergoing PSMA PET/CT stimulating forced diuresis through a combination of hydration and 10mg intravenous furosemide is an effective strategy to increase clearance of urinary activity and decrease peri-bladder artefacts, especially in ^18^F-DCFPyL PET/CT, whereas the benefit in ^68^Ga-PSMA-11 PET/CT is lower and should be balanced to a possible increase in urge complaints when longer scanning protocols are used to maintain image quality with limited availability of generator-produced ^68^Ga isotope. Based on the results of this study, forced diuresis using furosemide was reintroduced at our department for ^18^F-DCFPyL PET/CT.

## Conclusions

Increased urinary activity and incidence of peri-bladder artefacts were found in ^18^F-DCFPyL compared to ^68^Ga-PSMA-11 PET/CT. Effective reduction of urinary activity may be reached through forced diuresis using 10mg intravenous furosemide, which is especially advantageous in ^18^F-DCFPyL PET/CT.

## Data Availability

The datasets generated and analysed during the current study are not publicly available as these contain individual person’s data but are available from the corresponding author on reasonable request, after pseudonymization of the data and legal agreement.
